# X-linked hyper IgM syndrome with a novel CD40-ligand gene mutation suffered from infection of Talaromyces marneffei

**DOI:** 10.3389/fimmu.2026.1875082

**Published:** 2026-09-29

**Authors:** Juan Gu, Aiyan Ren, Jing Kong, Meiying Huang, Huijuan Jin, Chengxin Sun, Shiji Xiao, Enming Zhang, Zuochen Du

**Affiliations:** 1 Department of Pharmacy, Affiliated Hospital of Zunyi Medical University, Zunyi, China; 2 Department of Pediatrics, Affiliated Hospital of Zunyi Medical University, Zunyi, China; 3 Guizhou Children’s Hospital, Zunyi, China; 4 Nursing Department, Ordnance Industry Hygiene Research Institute, Xi’an, Shaanxi, China; 5 School of Pharmacy, Zunyi Medical University, Zunyi, China; 6 Guizhou Key Laboratory of Modern Traditional Chinese Medicine Creation, Zunyi Medical University, Zunyi, China; 7 Key Laboratory of Basic Pharmacology, Ministry of Education, Zunyi Medical University, Zunyi, China; 8 Department of Clinical Sciences in Malmö, Lund University Diabetes Centre, Lund University, Malmö, Sweden

**Keywords:** CD40L, gene mutation, gene sequencing, *Talaromyces marneffei*, the X-linked hyper-IgM

## Abstract

**Background:**

X-linked Hyper-IgM Syndrome (X-HIGM) is a primary immunodeficiency caused by pathogenic variants in *CD40L*. Loss of functional CD40 ligand interferes with T-cell–dependent B-cell responses and immunoglobulin class-switch recombination, resulting in susceptibility to recurrent and opportunistic infections. Because *CD40L* is located on the X chromosome, X-HIGM is usually diagnosed in males. Symptomatic females are rare, and the clinical and immunological features of these patients have not been well defined.

**Methods:**

We report a 4-year-old Chinese girl who developed disseminated Talaromyces marneffei infection and was subsequently diagnosed with X-HIGM. Her clinical history and laboratory findings were reviewed, and a comprehensive immunological evaluation was performed, including flow cytometric analysis. Genetic testing was carried out by next-generation sequencing, and the identified variant was confirmed by Sanger sequencing. The possible effect of the *CD40L* c.364 + 2T>A variant on RNA splicing was examined using the online AI tool RNA-Splicer. We also analyzed peripheral blood mononuclear cells (PBMCs) from the patient and an age- and sex-matched healthy control by single-cell RNA sequencing to further characterize abnormalities in peripheral immune-cell populations and their transcriptional profiles.

**Results:**

A previously undescribed heterozygous *CD40L* c.364 + 2T>A variant was identified in the patient and was confirmed to be *de novo*. Analysis of the PBMC transcriptomes showed differences across several immune-cell compartments. In particular, B-cell populations showed impaired programs related to maturation and immunoglobulin class switching, while alterations were also observed in T-cell developmental states. The proportions of several myeloid and dendritic-cell populations were reduced in the patient. In addition, cell–cell communication analysis indicated changes in interactions among multiple immune-cell subsets.

**Discussion:**

We identified a *de novo* splice-site variant in *CD40L*G in a symptomatic female child with X-HIGM and disseminated T. marneffei infection. The clinical presentation and single-cell transcriptomic findings highlight the broad effects of *CD40L* deficiency on the peripheral immune system. This case adds a previously unreported *CD40L*G variant to the spectrum of genetic changes associated with X-HIGM and provides additional information on the immune abnormalities that may occur in symptomatic female patients.

## 1 Introduction

X-HIGM is a prototypic inborn error of immunity characterized by impaired immunoglobulin class-switch recombination (CSR) and somatic hypermutation. Patients typically present with normal or elevated serum IgM, while IgG, IgA, and IgE are markedly decreased or absent (1, 2). The estimated prevalence is approximately 1 per 1,000,000 live births, and the median survival is about 25 years without hematopoietic stem cell transplantation (HSCT) (3, 4). X-HIGM is caused by mutations in *CD40L*(Xq26.3),which encodes CD154, a 32-kilodalton type II transmembrane glycoprotein composed of 261 amino acids and consists of a cytoplasmic tail, a transmembrane region, and an extracellular domain containing a tumor necrosis factor (THF) homology domain (5). CD154 is primarily expressed on activated T cells and platelets and plays a critical role in B-cell proliferation and immunoglobulin class-switching recombination (6, 7). CD40L-CD40 interaction is essential for B-cell activation, germinal center formation, myeloid cell maturation, and anti-fungal immunity. Disruption of this pathway results in combined humoral and cellular immunodeficiency, leading to recurrent bacterial, viral, fungal, and protozoal infections (8, 9).

*Talaromyces marneffei* is a thermally dimorphic fungus endemic to Southeast Asia and southern China (10, 11). It is a major opportunistic pathogen in immunocompromised individuals, including those with primary immunodeficiencies (12). Increasing evidence links severe T. marneffei infection to primary immunodeficiencies, including those caused by *CD40L* mutations (13–15).

Classically, X-HIGM affects males exclusively. Female carriers are usually asymptomatic unless extreme X-chromosome inactivation skewing occurs (16). Here, we describe a 4-year-old female with a novel *de novo* CD40L splice-site mutation (c.364 + 2T>A) presenting with disseminated T. marneffei infection. We used scRNA-seq to dissect the immune landscape and characterize the immune consequences associated with this rare variant.

## 2 Materials and methods

### 2.1 Subjects and sample collection

For Single-Cell RNA Sequencing, six months after the completion of antifungal treatment, the 4-year-old Chinese girl with primary immunodeficiency and a history of disseminated T. marneffei infection was followed up and re-evaluated. An age- and sex-matched healthy child served as control.

### 2.2 Next-generation sequencing and sanger sequencing

Genomic DNA was extracted from peripheral blood leukocytes using the Qiagen FlexiGene DNA Kit. A targeted panel covering 679 primary immunodeficiency-related genes was sequenced on the Illumina NextSeq 500 platform(Illumina, USA). Bioinformatics analysis included alignment, variant calling, and pathogenicity prediction using SIFT, PolyPhen-2, and MutationTaster2. Candidate variants were verified by Sanger sequencing.

### 2.3 Flow cytometric analysis of CD40L expression

The expression of CD40L was detected by flow cytometry in patients, her parents, and age-matched healthy control children. First, peripheral blood mononuclear cells (PBMCs) are isolated by density gradient centrifugation using lymphocyte separation medium. Add 1×10^6^ PBMCs to 500 μL RPMI 1640 medium containing 10% fetal bovine serum, 500 ng/mL ionomycin, and 50 ng/mL PMA, then culture at 37 °C for 4 hours. Subsequently, the cells were incubated at 4 °C for 30 minutes after adding 1 μL each of PerCP-anti-human-CD3 (300326), Brilliant Violet 510-anti-human-CD8a (301048), PE-anti-human-CD154 (310805), and PE-anti-human-IgG1 (MOPC-21). The final samples were analyzed using a FACS Canto Plus flow cytometer (BD Biosciences), with data processing performed using Flow Jo software (BD Biosciences). The percentage of CD3^+^CD8^-^CD154^+^ cells was represented by dot plot gating and compared with cells stained with isotype control reagents (CD3^+^CD8^-^IgG^+^). All antibodies were purchased from BioLegend and staining procedures were performed according to the manufacturer’s experimental protocols.

### 2.4 Microbiological and histopathological examination

Blood, bone marrow, pleural fluid, gastric fluid, and lymph node specimens were cultured for Talaromyces marneffei. Lymph node biopsy samples were subjected to periodic acid-Schiff (PAS) and Gomori methenamine silver (GMS) staining for fungal detection, as well as hematoxylin and eosin (H&E) staining to evaluate germinal center architecture.

### 2.5 Single-Cell RNA sequencing and bioinformatics analysis

Two months after completing antifungal therapy for Talaromyces marneffei infection, followed by a six-month post-treatment period, peripheral blood mononuclear cells (PBMCs) from this patient and a age- and sex-matched healthy control were analyzed via scRNA-seq.scRNA-seq libraries were prepared using the 10x Genomics Chromium Next GEM Single Cell 5′ Reagent Kits v2.0. TCR and BCR V(D)J libraries were enriched and sequenced on the Illumina NovaSeq 6000 platform. Raw data were processed with Cell Ranger v7.0.1 (GRCh38 reference). Downstream analysis was performed in R using Seurat v4.0.0, including quality control, normalization, batch correction (MNN), clustering, UMAP visualization, differential expression, and functional enrichment (GO/KEGG). Cell-cell communication was analyzed using CellChat.

### 2.6 In silico splicing prediction

The potential effect of the novel CD40L c.364+2T>A splice-site variant on pre-mRNA splicing was evaluated using the online AI-powered splicing prediction tool RNA-Splicer (Rare Disease Data Center, available at: https://rddc.tsinghua-gd.org/zh/tool/rna-splicer). The reference transcript NM_000074.3 was used for variant annotation and splicing pattern simulation. Wild-type and mutant splicing patterns were compared to predict abnormal splicing events, including splice-site gain/loss and potential frameshift alterations.

## 3 Results

### 3.1 Clinical presentation and antifungal treatment

A 4-year-old Chinese girl was admitted with 4-week abdominal pain and a several-day cough. Physical examination revealed abdominal distension and generalized lymphadenopathy. Chest and abdominal CT revealed extensive pulmonary lesions, mediastinal and abdominal lymphadenopathy, hepatic lymphatic congestion, and pelvic fluid (**Figures 1A, B**). Laboratory tests showed leukopenia, lymphopenia, elevated CRP (139.95 mg/L) and ESR (47 mm/h), markedly decreased IgG, and undetectable IgA (see **Supplementary Data Sheet 1** sheet laboratory tests for detailed Laboratory results).

**Figure 1:**
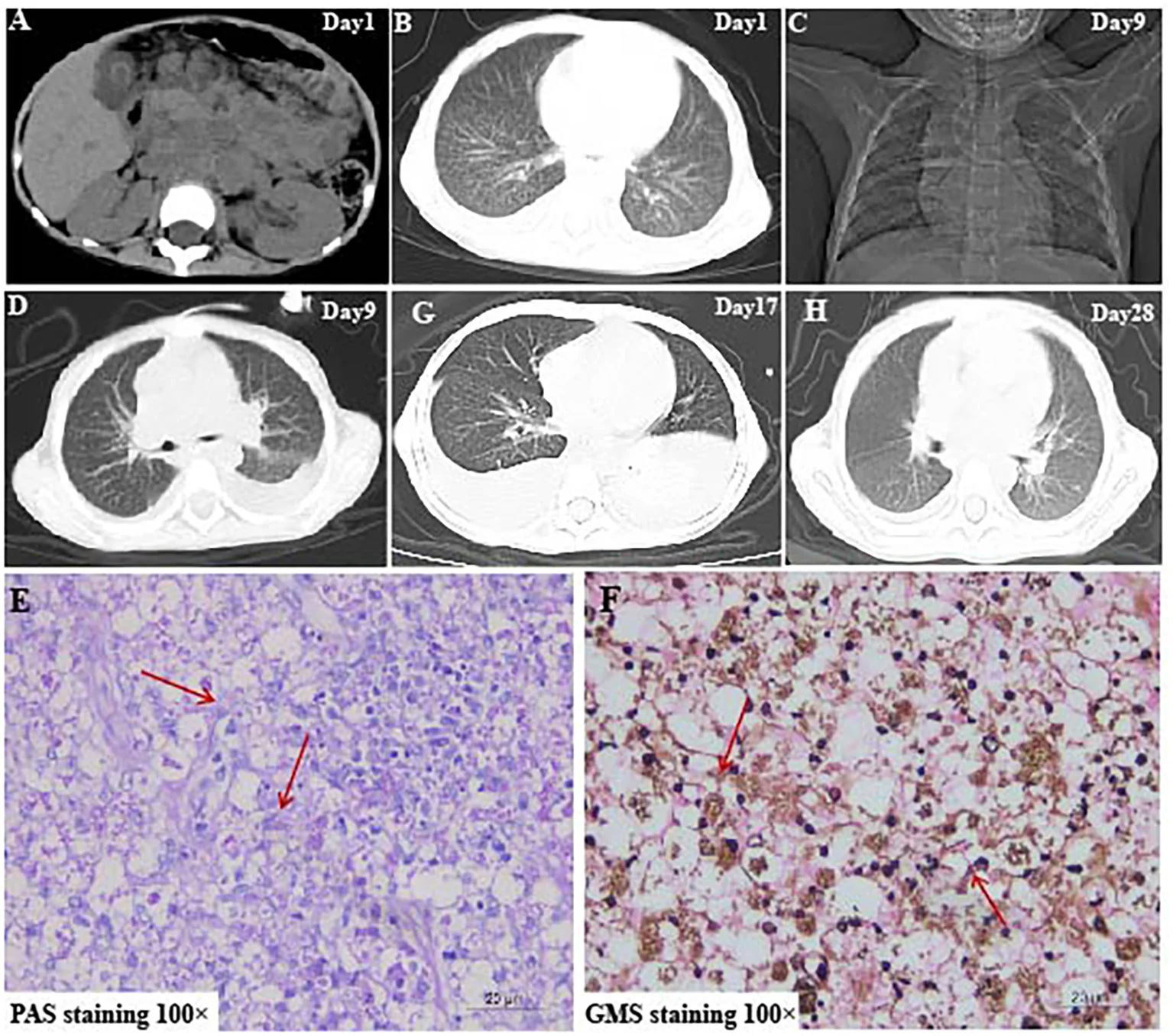
Dynamic radiological changes and fungal histopathology in the patient. **(A)** Chest CT on admission (day 1) showed extensive pulmonary infiltrates and mediastinal lymphadenopathy. **(B)** Abdominal CT on admission (day 1) demonstrated abdominal lymphadenopathy, hepatic lymphatic congestion, and pelvic effusion. **(C, D)** Chest radiograph **(C)** and chest CT **(D)** obtained on day 9 showed progressive enlargement of mediastinal lymph nodes and diffuse pulmonary infiltrates. **(E)** (PAS staining, 100×, scale bar = 20 μm): Red arrows indicate Talaromyces marneffei. Numerous 2~6 μm oval or sausage-shaped yeast cells with characteristic septa are present in lymph node tissue. The polysaccharide-rich fungal cell walls are strongly positive for magenta staining. Yeast cells are frequently clustered within macrophages, accompanied by inflammatory cell infiltration. **(F)** (GMS staining, 100×, scale bar = 20 μm): Red arrows indicate Talaromyces marneffei. Abundant 2-6 μm oval or sausage-shaped yeast cells with characteristic septa are seen in lymph node tissue. Fungal cell walls are stained dark brownish-black, with clear outlines and sharp contrast against the pale blue host cell nuclei counterstained with hematoxylin. **(G)** Chest CT on day 7 of antifungal therapy showed transient worsening of pulmonary inflammation. **(H)** Chest CT on day 28 of antifungal therapy revealed marked radiological improvement.

Initial antibacterial and anti-tuberculosis therapies showed no clinical response. Chest radiography and follow-up CT revealed progressive enlargement of mediastinal lymph nodes and diffuse pulmonary infiltrates (**Figures 1C, D**). *Talaromyces marneffei* was first isolated from blood cultures obtained on day 3 and confirmed after 7 days of anti-tuberculosis treatment; the pathogen was subsequently identified in cultures of bone marrow, pleural fluid, and lymph node specimens. Lymph node biopsy confirmed fungal infection by Periodic Acid-Schiff (PAS) and Gomori Methenamine Silver (GMS) staining (**Figures 1E, F**).

Antifungal therapy was immediately initiated with liposomal amphotericin B, gradually dose-escalated from 0.1 mg/kg/day to 2 mg/kg/day over 5 days to reduce adverse effects. After 16 days of intravenous liposomal amphotericin B, step-down therapy with oral itraconazole solution (2.5 mg/kg every 12 hours) was started on day 25, for a total antifungal course of 10 weeks. Fever resolved within 4 days. Although chest CT showed transient worsening of pulmonary inflammation on day 7 (**Figure 1G**), subsequent imaging demonstrated significant improvement (**Figure 1H**). The patient was discharged in stable condition.

### 3.2 Identification of a Novel *De Novo* CD40L mutation

Targeted sequencing identified a heterozygous *de novo* CD40L splice-site mutation(c.364 + 2T>A) in the patient, which was not detected in either parent (**Figure 2A**). In silico analysis with Mutation Taster2 predicted that this variant disrupts the canonical splice donor site, and may lead to aberrant mRNA splicing (17). Further analysis using an AI-based tool from the Rare Disease Data Center suggested that the mutation may generate two distinct aberrant alternative splicing isoforms (**Figure 2B**) (18). Flow cytometric analysis revealed markedly diminished CD154 expression on activated CD4+ T cells (**Figure 2C**). Given the indispensable role of CD40-CD40L signaling in germinal center formation (19, 20), hematoxylin and eosin (H&E) staining of the patient’s lymph node showed a complete absence of germinal centers (**Figure 2D**), supporting impaired CD40L function.

**Figure 2:**
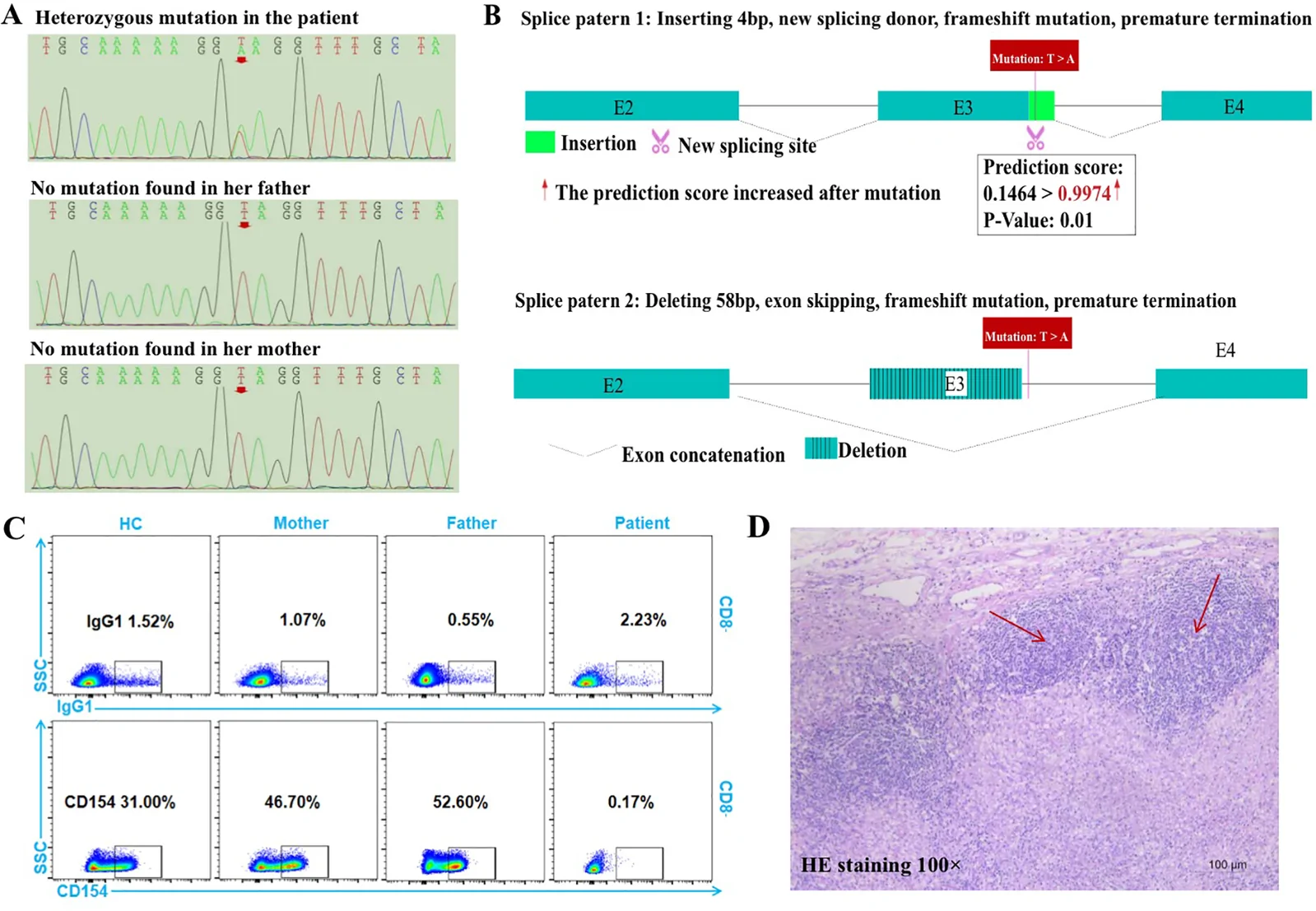
Identification and validation of a novel *CD40L* mutation and its impact. **(A)** Sanger Sequencing targeting CD40L identified a heterozygous *de novo CD40L* splice-site mutation(c.364 + 2T>A) in the patient, which was not detected in either parent. **(B)** In silico analysis with Mutation Taster2 predicted that this variant disrupts the canonical splice donor site, and may lead to aberrant mRNA splicing. **(C)** Flow cytometric analysis revealed markedly diminished CD154 expression on activated CD4+ T cells. **(D)** hematoxylin and eosin (H&E) staining of the patient’s lymph node showed a complete absence of germinal centers.

### 3.3 Abnormalities of B-cell clonotype expansion and immunoglobulin deficiency revealed by BCR sequencing

Following the single-cell RNA sequencing protocol detailed in the Methods section, we initially captured 14,257 cells (49.97%) from the patient and 14,558 cells (50.03%) from the control. After a second round of quality control filtering to exclude doublets, 19,184 high-quality cells were retained for downstream analysis, including 9,559 cells (49.83%) from the patient and 9,625 cells (50.17%) from the control.

PCA and UMAP analyses were used to characterize the B-cell populations and compare their distribution between the patient and control. The BCR repertoire showed a marked difference between the two samples. In the patient, class-switched heavy-chain clonotypes were largely absent. IGHA1 and IGHA2 were barely detected, while the IGHG1–IGHG4 isotypes were also much less abundant than in the control (**Figure 3A**). By contrast, IGHM represented a much larger proportion of the patient repertoire. IGHD was also detected at a higher relative level than in the control. This pattern was consistent with a predominance of unswitched B-cell receptors in the patient.

Changes were also observed in the light-chain repertoire. IGKC clonotypes were less abundant in the patient, as was IGLC1. In contrast, IGLC2 accounted for a larger proportion of the repertoire in the patient than in the control (**Figure 3A**). Thus, the changes in light-chain usage were not uniform across the different subclasses.

The differences became more apparent when BCR clonotypes were examined according to B-cell subset. Naive B cells accounted for most of the clonotypes detected in the patient, whereas memory B1, memory B2, switched B cells, and plasma cells contributed relatively few clonotypes (**Figure 3B**). The same pattern was evident from the cellular composition analysis. Although the overall B-cell compartment was represented in both samples, the patient had a predominance of naive B cells, with very limited representation of memory B cells, switched B cells, and plasma cells (**Figure 3C**). In comparison, these more differentiated populations were readily detected in the control.

Trajectory analysis provided additional evidence of differences in the organization of the B-cell compartment (**Figure 3D**). The control sample contained cells distributed across several B-cell states, including naive, memory B1, memory B2, switched B-cell, and plasma-cell populations. In the patient, the later B-cell populations were sparsely represented, in agreement with the BCR repertoire and compositional analyses. Taken together, the data indicate a substantial shift toward the naive B-cell compartment in this patient, accompanied by a marked reduction in memory, class-switched, and plasma-cell populations. These changes are consistent with impaired progression of B-cell differentiation and limited immunoglobulin class switching.

**Figure 3:**
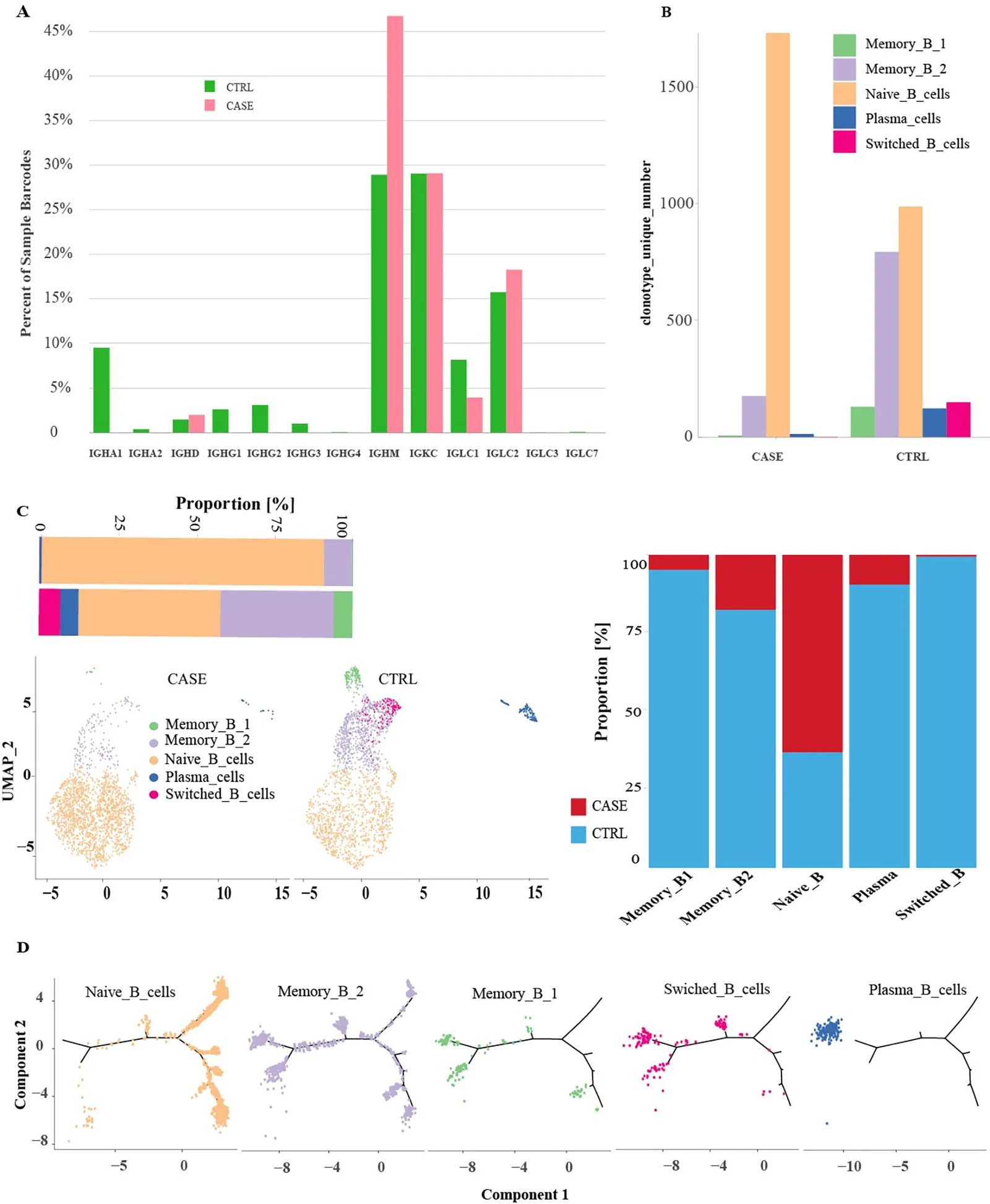
B-cell receptor repertoire, subset distribution, and differentiation trajectory are severely disrupted in the patient. **(A)** Altered immunoglobulin isotype and light-chain repertoire in the patient. **(B)** Abnormal B-cell subset distribution with impaired class switching and differentiation. **(C)** Altered composition of B-cell subsets compared with the control. **(D)** Disrupted B-cell differentiation trajectory by pseudotime analysis.

### 3.4 Composition of immune cell populations and subsets identified by single-cell RNA sequencing

We next examined the distribution of the major immune-cell populations and their subsets. The overall proportion of T cells was similar in the patient and control samples (**Figure 4A**), but their subset composition differed considerably. Naive CD4 and CD8 T cells were more abundant in the patient, as was the γδ T2 population. In contrast, memory CD4 T cells, regulatory T cells (Tregs), cytotoxic CD8 T cells, and γδ T1 cells were less represented (**Figure 4B**).

**Figure 4:**
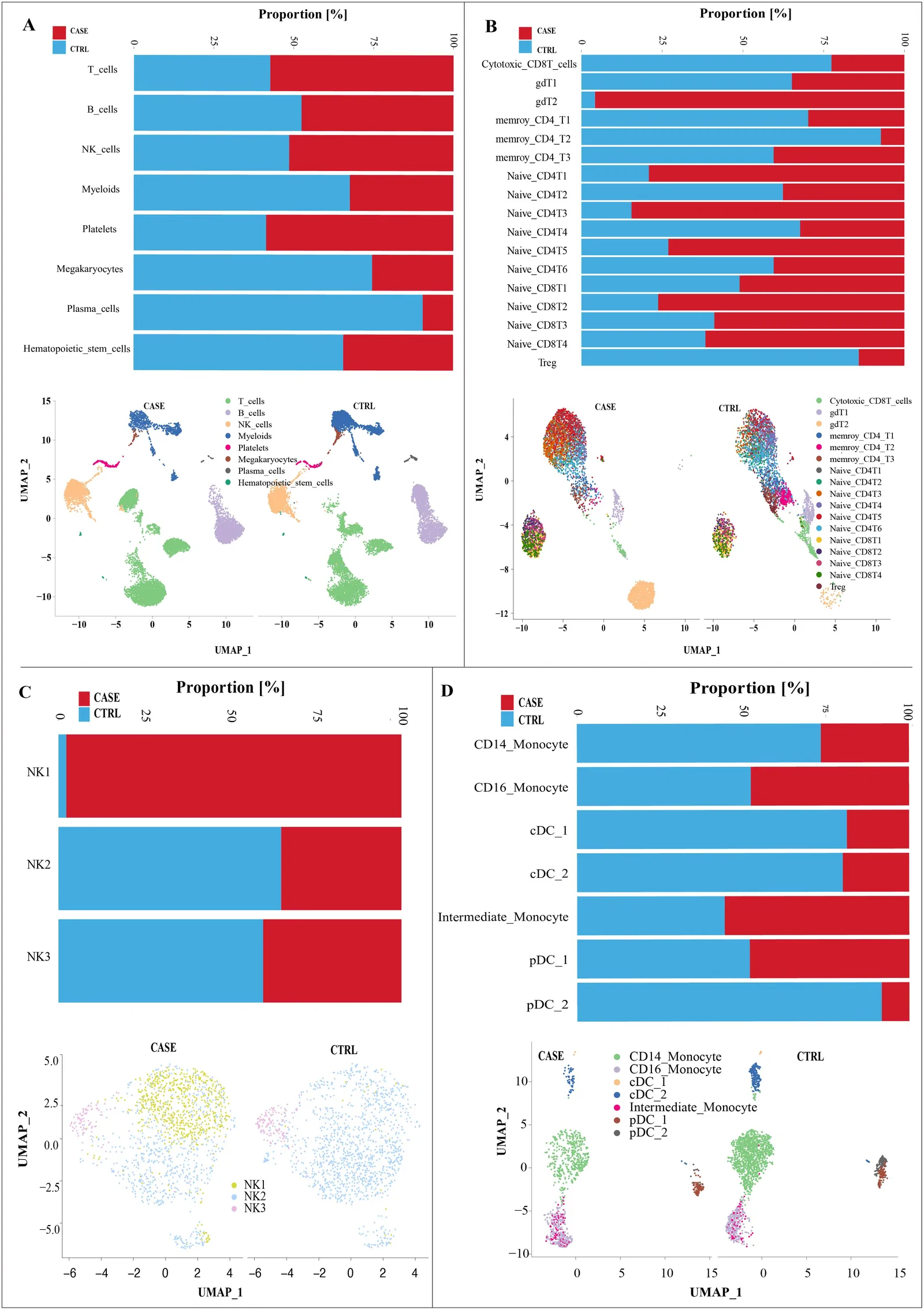
Profound subset-specific alterations in T, NK, and myeloid cell populations. **(A)** Comparable total T-cell and NK-cell counts between the patient and control. **(B)** Aberrant subset distribution of T cells and γδ T cells in the patient. **(C)** Skewed NK-cell subset composition with expanded NK1 cells. **(D)** Significantly reduced myeloid cell counts with subset-specific depletion.

The total number of NK cells was also comparable between the two samples (**Figure 4A**). At the subset level, however, NK1 cells accounted for a much larger proportion of the NK-cell compartment in the patient, whereas NK2 and NK3 cells were less abundant (**Figure 4C**). A different pattern was observed among myeloid cells. The total myeloid-cell population was smaller in the patient, with reductions in CD14 monocytes, cDC1, cDC2, and pDC2. CD16 monocytes, intermediate monocytes, and pDC1 showed less pronounced differences between the two groups (**Figure 4D**). The cell counts for individual subsets are provided in Supplementary data sheet 1 sheet cell counts, and the marker genes used for subset identification are listed in Supplementary data sheet 1 sheet cell type calssified markers. Differentially expressed genes with a fold change >1.5 for the respective cell populations are also provided in Supplementary data sheet 1 sheet difference fold changes >1.5.

The marked differences in the NK1 and γδ T2 populations prompted us to examine their transcriptional profiles in greater detail. GSVA of NK1 cells showed enrichment of gene sets related to leukocyte-mediated immunity, the alternative complement pathway, and antigen processing and presentation. Genes associated with inhibitory cell-surface receptor signaling were also enriched. In contrast, gene sets related to translation, ribosomal functions, and mRNA catabolism showed lower activity. Of particular interest, the gene set corresponding to the defense response to fungus (GO:0050832) had a strongly negative GSVA score in NK1 cells, suggesting reduced activity of antifungal response-related transcriptional programs in this subset.

The γδ T2 cells showed a different transcriptional pattern. Gene sets associated with NK-cell chemotaxis, regulation of lymphocyte chemotaxis, cytolysis, and NK-cell-mediated immunity were enriched. Several pathways related to immune regulation were also increased, including negative regulation of microglial activation, macrophage apoptosis, and chemokine signaling. Unlike NK1 cells, γδ T2 cells did not show a significant change in the defense response to fungus gene set (GO:0050832) (**Figure 5**). These results suggest that the two expanded lymphocyte populations were transcriptionally distinct despite both being enriched in the patient.

**Figure 5:**
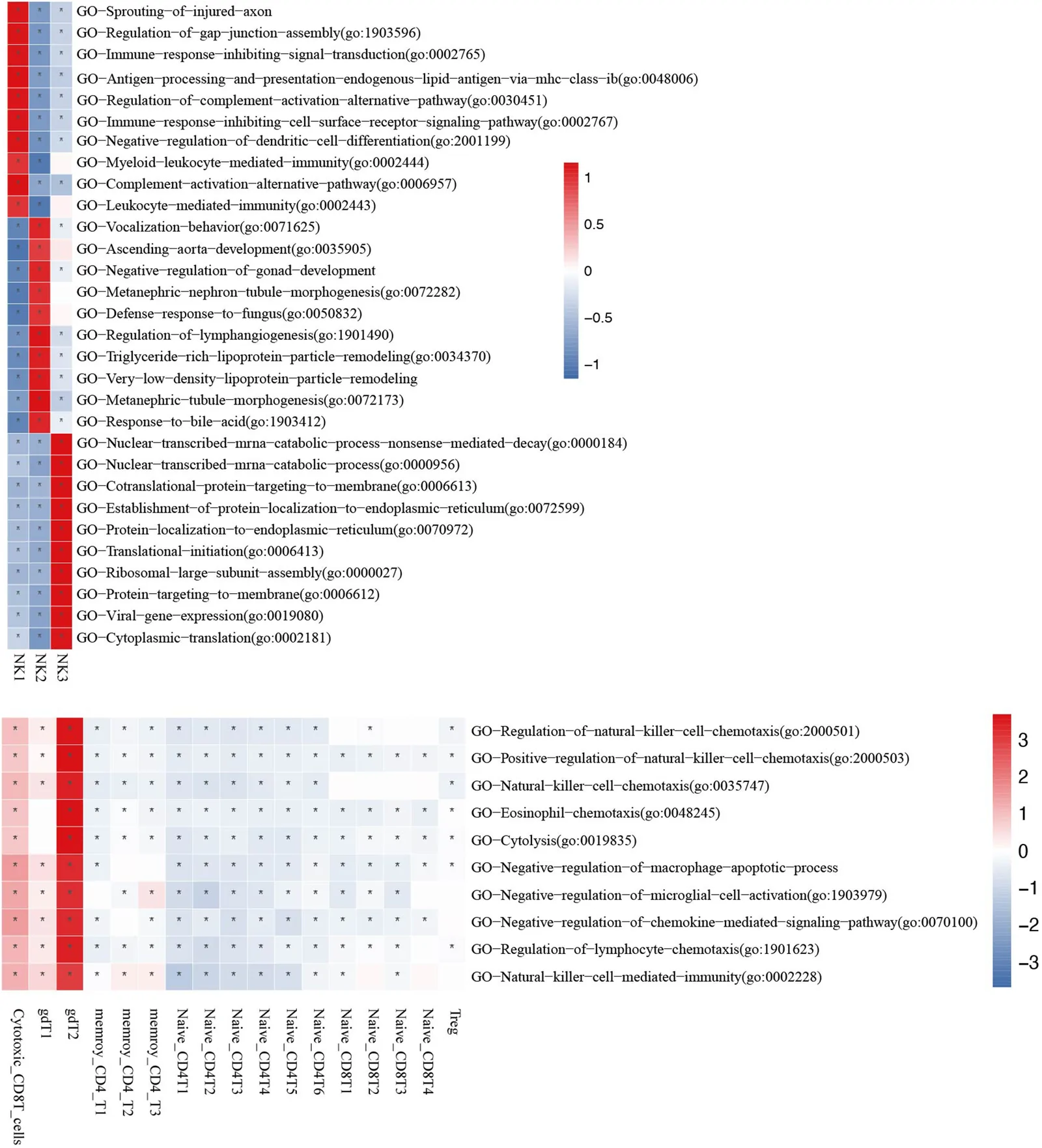
Enhanced immunoregulatory functions in expanded NK1 and γδ T2 subsets GSVA was performed to explore the functional characteristics of NK1 and γδ T2 subsets. In Upper panel, NK1 cells exhibited significant upregulation of immune response-inhibiting signaling pathways, indicating an immunoregulatory phenotype. In Lower panel, γδ T2 cells displayed prominent upregulation of pathways related to NK cell chemotaxis and activation, demonstrating a strong capacity to modulate NK cell function, in contrast to other T cell subsets. Red indicates high pathway enrichment, and blue indicates low enrichment; asterisks denote statistically significant differences (P < 0.05).

We then used CellChat to examine potential changes in ligand–receptor communication among the immune-cell populations. Compared with the control, the patient sample contained fewer inferred interactions, together with a lower overall interaction strength (**Figure 6**). Thus, the immune-cell network in the patient was not simply altered in terms of cell composition; the inferred communication landscape was also different.

**Figure 6:**
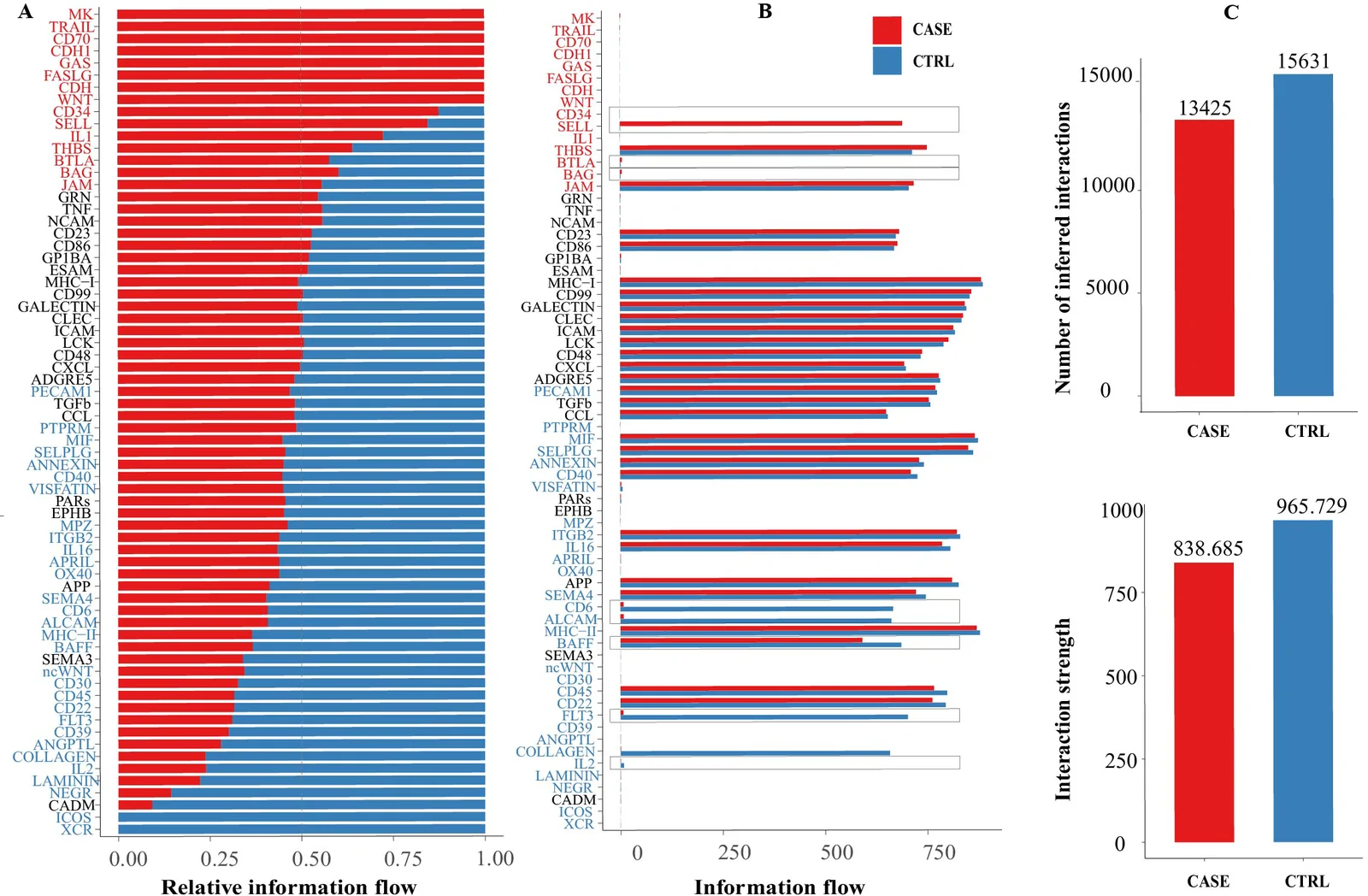
Global reduction and remodeling of intercellular communication networks in the patient. **(A)** Relative information flow of key ligand–receptor pairs in intercellular communication in CASE and CTRL. **(B)** Information flow of major signaling pathways in CASE and CTRL. **(C)** Quantitative comparison of total intercellular communication information flow in CASE and CTRL.

At the pathway level, several signaling systems were relatively more prominent in the patient, including MK, TRAIL, CD70, CDH1, GAS, FASLG, WNT, and SELL. Other pathways showed lower activity, including ICOS, XCR, CADM, NEGR, LAMININ, COLLAGEN, ANGPTL, FLT3, CD22, and CD45. The reduction in ICOS- and XCR-related interactions is compatible with changes in T-cell co-stimulatory and dendritic cell–lymphocyte communication. Similarly, the lower activity of LAMININ- and COLLAGEN-associated signaling may reflect differences in interactions involving the extracellular matrix and the immune-cell microenvironment.

Taken together, the single-cell data revealed differences in both immune-cell composition and the inferred communication network in this patient. The most notable changes included expansion of naive T-cell, NK1, and γδ T2 populations, loss of several myeloid and dendritic-cell subsets, and marked reduction of memory and effector populations. The GSVA and CellChat analyses further showed that these cellular changes were accompanied by alterations in antifungal-response programs and immune-cell communication, providing a broader view of the immune abnormalities associated with CD40L deficiency in this patient.

## 4 Discussion

This case describes a 4-year-old girl with X-HIGM who developed disseminated Talaromyces marneffei infection and was found to carry a heterozygous splice-site variant in CD40LG. The combination of clinical findings, genetic testing, immunological assessment, and single-cell RNA sequencing allowed us to examine the disease from several perspectives. In addition to identifying a previously unreported CD40LG variant, the findings illustrate the extensive changes in peripheral immune-cell composition and function associated with CD40L deficiency. The case also draws attention to the difficulties in recognizing talaromycosis in children with an underlying inborn error of immunity.

The diagnosis of T. marneffei infection can be particularly challenging in areas where tuberculosis is common. In the present case, the initial clinical presentation and imaging findings led to a diagnosis of tuberculosis, resulting in a delay in appropriate antifungal treatment. Pulmonary and systemic manifestations of talaromycosis can overlap with those of tuberculosis and other severe infections, especially in patients with impaired cellular immunity. The subsequent dissemination of infection in our patient illustrates the consequences of this diagnostic overlap. For HIV-negative children with severe, recurrent, disseminated, or otherwise unusual infections, T. marneffei should therefore be considered when clinically appropriate, and the possibility of an underlying primary immunodeficiency should not be overlooked.

One feature of the clinical course was the absence of obvious hypotension despite evidence of a systemic inflammatory response. CD40L is involved in T-cell–dependent activation of antigen-presenting cells and in the subsequent production of inflammatory mediators. Circulating soluble CD40L has also been associated with disease severity in sepsis (21). Whether impaired CD40-CD40L signaling alters the inflammatory response sufficiently to influence hemodynamic instability remains uncertain. Although the relatively stable blood pressure in this patient raises this possibility, a causal relationship cannot be inferred from a single case. Further clinical observations and experimental studies will be needed to determine whether CD40L deficiency affects the inflammatory and cardiovascular manifestations of severe infection.

The response to antifungal treatment also deserves attention. After escalation of liposomal amphotericin B, the patient's fever subsided, while pulmonary lesions on imaging temporarily appeared to progress. A discrepancy between clinical improvement and radiological evolution has been described during treatment of invasive fungal infections. In this setting, radiological worsening does not necessarily indicate microbiological progression or treatment failure and may partly reflect persistent or evolving inflammatory changes within previously infected tissues. Clinical status, microbiological findings, and serial imaging should therefore be considered together when assessing the early response to antifungal therapy.

The immunological findings were in keeping with the established role of CD40L in B-cell activation and immunoglobulin class-switch recombination. Interaction between CD40L on activated T cells and CD40 on B cells is required for germinal-center formation, antibody class switching, and the development of memory B cells. Loss of this signaling pathway typically results in an immunological profile characterized by preserved or increased IgM together with reduced IgG and IgA. Our patient showed the same general pattern. The single-cell and BCR analyses provided additional detail: naive B cells were relatively predominant, whereas memory and class-switched B-cell populations were markedly reduced, with very few switched B-cell and plasma-cell clonotypes detected. These observations are compatible with impaired progression of B cells toward more differentiated and class-switched states and provide a cellular context for the abnormal immunoglobulin profile observed in this patient. 

CD40L plays a critical role in immunoglobulin class-switch recombination, germinal center formation, and memory B-cell generation. Disruption of this pathway leads to the characteristic immunological features of X-HIGM, including elevated IgM and reduced IgG and IgA levels. Our findings are consistent with this mechanism, as demonstrated by immunophenotyping and single-cell transcriptomic data showing near-complete loss of class-switched memory B cells. Structural and in silico analyses further suggest that the predicted splice alteration may disrupt the extracellular domain required for CD40 binding, thereby potentially impairing receptor-ligand interaction (18, 22).

Notably, the patient was heterozygous for the CD40L mutation, which is atypical for an X-linked recessive disorder. Although female carriers are usually asymptomatic, skewed X-chromosome inactivation has been reported to result in reduced CD40L expression and clinical manifestations (16). A dominant-negative mechanism represents an alternative and highly plausible explanation for the full disease phenotype in this heterozygous female patient. Su et al. demonstrated that CD40L variants lacking the TNF homology domain (such as those resulting from exon 3 skipping) can bind to the wild-type protein via the stalk region, despite the absence of the trimerization domain. This binding impairs the maturation and transport of the wild-type protein to the cell surface, exerting a dominant-negative effect (23). The c.364 + 2T>A variant identified in our patient is strongly predicted to disrupt splicing based on in silico analysis, and it is therefore possible that this variant behaves in a similar manner.

However, formal proof of this mechanism would require experimental demonstration that: (i) X-chromosome inactivation is random (thereby excluding skewed inactivation as the sole cause of the phenotype), and (ii) the aberrantly spliced transcript is expressed at the RNA level and is not readily degraded by nonsense-mediated decay (NMD), with characterization of the relative abundance of full-length and aberrantly spliced isoforms. If the aberrant transcript were readily degraded, the dominant-negative hypothesis would be invalidated; conversely, if it is present in quantities comparable to the wild-type transcript, the probability of a dominant-negative effect would be substantially higher. These validations were not performed in the present study. Therefore, while the dominant-negative effect remains a compelling hypothesis, it should be regarded as a plausible but unproven speculation. Alternative mechanisms, such as extreme skewing of X-inactivation (which cannot be formally excluded) resulting from complete loss of the wild-type allele’s expression, may also contribute to the observed phenotype. Further studies using RNA sequencing together with quantitative assessment of allele-specific expression will be needed to determine how the variant affects CD40LG transcript processing and expression.

The single-cell RNA-sequencing data showed abnormalities across both adaptive and innate immune-cell compartments. The B-cell compartment was characterized by fewer memory B cells and plasma cells and a relative predominance of naive B cells. This pattern is compatible with impaired B-cell maturation and class switching, which is expected from disruption of the CD40-CD40L pathway (18, 7). Within the T-cell compartment, naive populations were relatively increased, whereas memory T cells and regulatory T cells were less abundant. These differences may reflect altered T-cell activation or maturation in the setting of defective CD40-CD40L-dependent immune interactions (24). 

Changes were not restricted to lymphocytes. The patient had fewer CD14 monocytes as well as reductions in several dendritic-cell subsets. Because CD40-CD40L interactions contribute to communication between T cells and antigen-presenting cells, these changes may have consequences for antigen presentation and subsequent immune activation (25–27). Whether the altered myeloid-cell composition directly contributed to the patient's susceptibility to opportunistic infection, however, cannot be determined from the present data.

The NK-cell and γδ T-cell findings were of particular interest. Six months after treatment for T. marneffei infection, the patient had a higher proportion of NK1 and γδT2 cells than the control. The reason for this difference remains uncertain. It may represent, at least in part, a change in immune-cell composition during recovery from severe fungal infection, although a compensatory response to the underlying immunodeficiency cannot be excluded. The transcriptional profile of NK1 cells was also different from that of the other NK-cell populations. Gene sets related to leukocyte-mediated immunity, alternative complement activation, and antigen processing and presentation were enriched, whereas pathways associated with protein synthesis, ribosomal functions, and mRNA degradation were less active. Of note, the defense response to fungus gene set (GO:0050832) showed a negative GSVA score in NK1 cells. This observation is interesting in the context of the patient's history of disseminated T. marneffei infection, but it should be interpreted cautiously. In γδT2 cells, the enriched pathways were mainly related to cytolytic activity, cell migration, and immune regulation, including regulation of microglial activation and macrophage apoptosis. These transcriptional features may be relevant to immune homeostasis.

At the level of the immune-cell network, CellChat analysis showed fewer inferred ligand-receptor interactions and lower overall interaction strength in the patient than in the control. This finding points to a substantial change in the organization of intercellular communication. Several signaling pathways involved in immune-cell development and activity were reduced in the patient, including CD6–ALCAM, TNFSF13B–TNFRSF13C (BAFF–BAFFR), IL-7–(IL7R + IL2RG), and FLT3L–FLT3. CD6–ALCAM interactions contribute to immunological synapse formation and T-cell activation (28), whereas BAFF–BAFFR signaling supports the survival and maturation of peripheral B cells (29). IL-7 is an important regulator of T-cell development and homeostasis (30), and FLT3L–FLT3 signaling is involved in the differentiation of dendritic cells from hematopoietic progenitors (31). The reduction of these signaling pathways is therefore consistent with the lower representation of memory B cells, memory T cells, plasma cells, and dendritic-cell subsets observed in the patient.

A different pattern was seen for several other ligand–receptor pairs, including SELL–CD34, BTLA–TNFRSF14, and BAG6–NCR3, which showed increased interaction strength in the patient. The SELL–CD34 pair is involved in the early steps of lymphocyte trafficking, particularly the tethering and rolling of naive lymphocytes on high endothelial venules before their entry into secondary lymphoid tissues (32–34). 

Its increased activity may be related to the greater proportion of naive lymphocytes in the patient. BTLA–TNFRSF14 (HVEM) is an inhibitory signaling pathway that can limit T-cell activation and cytokine production (35), and its increased activity may further influence the altered adaptive immune-cell profile. BAG6–NCR3, involving the NKp30 receptor, has been implicated in NK-cell activation (36). The increase in this interaction is of interest in view of the expanded NK and γδ T-cell populations and the enrichment of cytotoxicity-related gene sets observed in our single-cell analysis.

Notably, elevated BAG6 expression in T cells may reflect a compensatory response to proteotoxic stress. BAG6 (also known as BAT3) functions as a key component of the cellular protein quality control machinery, facilitating the recognition, chaperoning, and delivery of misfolded or defective polypeptides to the ubiquitin-proteasome system (37, 38). Increased BAG6 expression in this context may reflect a broader cellular proteostasis response; however, whether aberrant CD40L protein products are generated from the splice-site variant remains unknown. Collectively, these findings indicate that CD40L deficiency disrupts not only individual immune cell compartments but also the broader intercellular communication networks required for coordinated immune responses, while simultaneously promoting compensatory activation of innate cytotoxic pathways.

We further observed reduced MHC-II expression, CD40-CD40L interactions, and multiple co-stimulatory signaling pathways (including ICOS, OX40, CD30), all of which are essential for optimal CD4^+^ T-cell help and B-cell activation (39–41). Consistently, diminished BAFF, APRIL, and FLT3 signalings correlated with the loss of memory B cells, plasma cells, and regulatory T cells, highlighting the critical role of these pathways in maintaining humoral and immune regulatory compartments (42–44). In addition, impaired CD45-CD22 signaling may predispose to immune dysregulation and autoimmunity, a recognized complication of primary immunodeficiencies involving class-switch recombination defects (45, 46).

Taken together, the data suggest that CD40L deficiency affects several components of the immune system. The abnormalities were not limited to individual immune-cell populations; changes were also observed in the inferred communication between different cell types, involving both adaptive and innate immune compartments.

### 4.1 Novelty and significance

This case adds several observations to the current understanding of X-HIGM, particularly with respect to female patients and severe fungal infection. 

A previously unreported heterozygous splice-site variant in CD40LG (c.364+2T>A) was identified in this patient and confirmed to be de novo. We searched available public databases, including HGMD, OMIM, and ClinVar, and did not identify a previous record of this variant. In silico analysis predicted an effect on RNA splicing. Together, these findings support the possibility that c.364+2T>A is a disease-causing variant, although functional studies will be required to establish its effect on CD40LG transcript processing and protein expression. The finding also adds a new variant to the known spectrum of CD40LG abnormalities.

The presentation in a female patient is another notable feature of this case. X-HIGM is classically inherited as an X-linked recessive disorder and is therefore predominantly recognized in males. Symptomatic females are uncommon. In our patient, the presence of a heterozygous CD40LG variant was accompanied by a clinical and immunological phenotype compatible with X-HIGM. One possible explanation is that the variant allele was preferentially expressed as a consequence of skewed X-chromosome inactivation. A dominant-negative mechanism has also been proposed as a possibility, but this cannot be established from the present data. Regardless of the underlying mechanism, this case illustrates that X-HIGM should not be excluded solely on the basis of female sex. In girls with severe or unusual infections accompanied by hypogammaglobulinemia or other findings suggestive of an inborn error of immunity, analysis of X-linked genes may therefore be warranted.

The single-cell RNA-sequencing analysis provided an additional view of the immune abnormalities in this patient. Changes were detected in both adaptive and innate immune compartments. Within the B-cell lineage, the reduction in memory and class-switched populations, together with the relative predominance of naive B cells, was consistent with the known defect in CD40-CD40L-dependent B-cell maturation. Differences were also observed among T-cell, monocyte, and dendritic-cell populations. In addition, CellChat analysis identified changes in several ligand-receptor communication pathways. Although these findings do not establish a direct mechanism for each cellular abnormality, they indicate that the immunological consequences of CD40L deficiency extend beyond impaired antibody class switching.

The occurrence of disseminated Talaromyces marneffei infection is also clinically relevant. In children without HIV infection, disseminated talaromycosis is uncommon and may be an important clue to an underlying inborn error of immunity. In the present case, recognition of the fungal infection and subsequent identification of the CD40LG variant were important for establishing the underlying diagnosis. Severe or disseminated T. marneffei infection, particularly when accompanied by unusual clinical features or poor response to standard treatment, should therefore prompt consideration of an underlying immunodeficiency. Establishing the genetic diagnosis may also influence long-term management, including decisions regarding antimicrobial prophylaxis, immunological monitoring, and hematopoietic stem cell transplantation (HSCT).

Overall, this case expands the clinical and genetic spectrum of X-HIGM by describing a symptomatic female patient with a previously unreported CD40LG splice-site variant and disseminated T. marneffei infection. The single-cell analysis further demonstrated abnormalities across multiple immune-cell compartments and their inferred communication networks. These observations emphasize the clinical value of considering X-HIGM in female patients with severe opportunistic infections and provide a basis for further investigation of the molecular consequences of the c.364+2T>A variant.

### 4.2 Limitations and strengths

This study has several limitations. First, due to the extreme rarity of CD40L mutations—particularly in female patients—our analysis was restricted to a single case with one age- and sex-matched healthy control, which may limit the generalizability of our findings. Second, the 5′-biased nature of single-cell RNA sequencing (scRNA-seq) constrains the accurate detection of full-length transcripts, making it difficult to definitively distinguish aberrantly spliced isoforms from wild-type transcripts. Third, cell-cell communication analyses are inferred from transcriptomic data and therefore may not fully recapitulate functional protein-level interactions, especially for immune mediators that are tightly regulated and induced only upon specific activation signals. Fourth, and importantly, RNA-based experimental validation of the predicted splicing defect (e.g., RT-PCR to detect the aberrantly spliced transcript) was not performed in this study. Similarly, formal analysis of X-chromosome inactivation status was not conducted, which limits our ability to definitively distinguish between dominant-negative effects and skewed X-inactivation as the underlying pathogenic mechanisms in this heterozygous female patient. Consequently, the possibility that the identified variant exerts a dominant-negative effect remains to be established. Further studies, particularly those assessing allele-specific expression and the functional consequences of the variant at the protein level, will be needed to clarify this mechanism.

Despite these limitations, our study has several notable strengths. The use of scRNA-seq allowed us to examine immune cell composition and transcriptional changes associated with CD40L deficiency at single-cell resolution. More importantly, we identified a previously unreported heterozygous CD40L variant in a female patient who presented with the characteristic clinical features of X-HIGM. This finding adds to the limited genetic evidence for CD40L deficiency in females and broadens the recognized clinical and genetic spectrum of this rare primary immunodeficiency. Taken together, our findings provide detailed immunological insights and may help inform further investigations into the mechanisms underlying CD40L-related immune dysfunction.

## Data Availability

The dataset generated and/or analyzed during the current study are not available to protect participant anonymity. Data access requests can be directed to the corresponding authors and will be considered on a case-by-case basis.
